# Collagen fibril diameter quantification using interference confocal reflectance microscopy

**DOI:** 10.1364/BOE.596950

**Published:** 2026-05-11

**Authors:** Eric Hall, Seyed Mohammad Siadat, Jeffrey Ruberti, Charles A. DiMarzio

**Affiliations:** 1 Northeastern University, Electrical and Computer Engineering, 360 Huntington Avenue, Boston, Massachusetts, USA; 2 Northeastern University, Bioengineering, 360 Huntington Avenue, Boston, Massachusetts, USA; 3 Northeastern University, Mechanical and Industrial Engineering, 360 Huntington Avenue, Boston, Massachusetts, USA

## Abstract

Understanding collagen fibril assembly and remodeling is critical to understanding the kinetics of assembling larger collagen structures. Being able to quantify a fibril’s mechanics while interacting with the collagen molecule would be a large step towards understanding how fibrils both self-assemble and remodel with larger collagen structures. *In-vitro* experimentation with individual collagen fibrils has opened the door to many possibilities for understanding collagen at the nanometer scale; however, many collagen fibrils have diameters that lie below the Abbe diffraction limit and therefore cannot be quantified with traditional microscopy methods. This presents a barrier for being able to quantify a fibril’s mechanical properties (stress, toughness) while simultaneously interacting with collagen monomers. The objective of this study was to design a collagen fibril diameter measurement method using confocal reflectance microscopy that could quantify fibril diameters below the diffraction limit, ideally well below 300 nm. In addition, the method should be able to integrate with confocal fluorescence systems, which are used to track collagen monomers and labeled collagen fibrils. This setup would allow real-time quantification of how collagen monomers interact with existing fibrils to grow and repair over time, while under cyclic loading. A 2D finite difference time domain (FDTD) simulation was first used to estimate the interference patterns at each excitation wavelength. Type 1 collagen fibrils (dry) were stretched over polydimethylsiloxane (PDMS) trenches, ∼25 µm wide, and imaged using a Zeiss LSM 880 confocal reflectance microscope. The selected wavelengths were 488 nm, 514 nm, and 561 nm. Fibrils were then imaged using a Hitachi S-4800 FE-SEM to estimate the range of fibrils imaged and used for assisting algorithm development. The interference pattern from the confocal reflectance imaging results matched the expected shape found in the 2D FDTD. These results were then fed through an algorithm developed with the FDTD to estimate the collagen fibril’s diameter, and directly overlaid with the registered scanning electron microscopy (SEM) images. Interference confocal reflectance microscopy (I-CRM) provides a promising modality for measuring collagen fibril diameters well below the diffraction limit. It provides several advantages over other methods, including non-lethality, no fluorescent staining, and a particularly promising ability for no-registration calibration.

## 1. Introduction

### 1.1. Collagen remodeling and quantification

Collagen is the most abundant protein in mammals and is the primary structural component of structures such as bone, ligament, skin, and sclera [1]. Its molecular unit (commonly referred to as a collagen monomer) is 1.5 nm in diameter and 300 nm in length [2–6]. These monomers then form collagen fibrils ranging in size from 20-500 nm, which then form larger collagen fibers and collagen macro-structures. These structures are resilient to mechanical loading and unloading on decade long time scales *in-vivo*, yet the mechanical, chemical and biological mechanisms on how this is achieved remain poorly understood [1,5].

Due to this, a large effort towards understanding and quantifying collagen fibril mechanics has emerged. In addition, the study of individual monomers and monomer assembly into fibrils can be done in-vitro, making *in-vitro* collagen fibril quantification a powerful tool towards understanding collagen assembly [7–13]. Many of these experiments involve the use of scanning electron microscopy (SEM) or atomic force microscopy (AFM), both of which are capable of sub 50 nm diameter resolution. A popular approach is to use SEM or AFM to measure collagen fibril diameters, then use optical techniques such as CRM or CFM to quantify other properties of fibrils/fibers [14–16]. While SEM is incompatible with live imaging, AFM has been used to study collagen fibril formation on substrate for diameter, D-banding, and mechanical property quantification [17–19].

However, these methods have not been demonstrated with simultaneous and continuous imaging with collagen monomers via fluorescence microscopy, away from a substrate. Therefore the direct interaction between existing fibrils and monomers cannot be studied. This presents an opportunity for high-magnification optical microscopy as a high-resolution, non-destructive alternative for quantifying both collagen monomers and fibril diameters. The former typically is investigated with fluorescent tags [,^4^,^20^], while the latter can be investigated using endogenous contrast created by the refractive index changes caused by a collagen fibril [3,21].

Techniques such as differential interference contrast edge intensity shift (DIC-EIS) [3,21] or quantitative optical tweezers microscopy [22] attempt to use optical interference methods quantify the fibril diameter. DIC-EIS is a method capable of quantifying collagen fibril diameter between 100-300 nm, with accuracies of 11 nm and 4 nm for dehydrated and hydrated fibrils, respectively [3]. Despite efforts to enhance DIC-EIS [21], this method has some noteworthy disadvantages including difficulty integrating with fluorescence microscopy, unknown performance below 70 nm, and the requirement to calibrate with a higher magnification microscope (either SEM or AFM).

This paper discusses interference confocal reflectance microscopy (I-CRM) as an alternative to current methods for quantifying collagen fibril diameter. Quantifying diameter is critical to studying the mechanics of individual fibrils [9,23], however the ability to integrate with monomer detection techniques (and therefore fluorescence microscopy) is critical to understanding how monomer dynamics effect fibril growth. Confocal reflectance microscopy has already been shown to be useful in imaging collagen fibrils by *Yang* [14,24]. In addition, *Yang et. al* has shown that CRM and confocal fluorescence microscopy (CFM) can be used harmoniously [14,24].

Since I-CRM is capable of integrating seamlessly with CFM, we can provide diameter AND monomer information in near real time, allowing for longer term studies of collagen assembly up to several days. Labeled collagen monomers are used to track the orientation of individual monomers and assembled fibrils, and being able to track this orientation and the overall fibril diameter simultaneously could further our understanding of how monomers orient, attach, and align with already existing collagen fibrils [20]. The DIC-EIS method discussed previously cannot simultaneously track collagen monomers, due to being a transmission broadband method. In addition, I-CRM is capable of making individual pixel esitmations of collagen fibril diameters

The second and unique advantage of CRM is its ability to take advantage of local interference. This phenomenon occurs when a coherent source reflects from two surfaces of an object that are slightly separated, such as a thin-film. This subset of microscopy is known as interference reflectance microscopy (IRM), reflectance interference contrast microscopy (RICM), and most specifically in this case interference confocal reflectance microscopy (I-CRM). All of these methods differ from other interference imaging methods such as optical coherence tomography (OCT) or optical coherence microscopy (OCM), which require a dedicated reference line and complicates the optical train of the microscope.

I-CRM already has a background in soft-tissue applications, more specifically quantifying intra-cellular distances and cell adhesion [25]. Our work is unique in quantifying structures where the object itself has a lateral size well below the size of the excitation beam, such as a collagen fibril. In this study, we show that CRM can not only visualize collagen fibrils well below the diffraction limit, but can also harness its local interference ability to measure collagen fibril diameter both with and without SEM calibration. We also feel that due to I-CRM’s non-reliance on imaging orientation and ability to visualize small changes in fibril diameter, it has a future as a full field of view (FOV) microscopy method rather than just a diameter quantification modality.

### 1.2. Working principle

I-CRM’s working principle is identical to IRM, RICM, and the traditional thin-film problem. In the simplest approximation, with collagen fibrils having a refractive index of approximately $n_{fibril}=1.41$ [26], the first two reflections, caused by the front and back surfaces of the fibril, will interfere constructively and destructively to modulate the amount of backscattered light according to $\frac{2\pi }{\lambda }\times OPD$ , where λ is the excitation wavelength of the confocal source and OPD is the optical path difference,

(1) $$OPD=2\times n_{fibril}\times d_{fibril}$$

for a fibril diameter of $d_{fibril}$ . Thus, the solution of the sinusoidal equation for $d_{fibril}$ is non-unique. Because the sinusoid’s period depends on λ , excitation with N different wavelengths can remove ambiguities, with the N different sinusoids as parametric functions of $d_{\lambda }$ forming a spiral in an N –dimensional signal space [25].

For the current problem, the approximation of the above equations is not sufficient. First, a confocal microscope source is typically a diffraction-limited Gaussian beam focused near the object rather than a plane wave. Second, the collagen fibrils for this study lie near or below the expected size of that diffraction-limited focal diameter. Finally, the wavefront and the fibril surfaces are both curved. These departures from the simple approximation make theoretical modeling more challenging, although we still expect a similar periodicity.

For these reasons, a numerical approach such as Mie scattering or finite difference time domain (FDTD) was needed. Due to FDTD’s ability to simulate multiple fibril geometries and common imaging situations (such as a collagen fibril on glass), FDTD was chosen for this study. The following sections describe how FDTD was used to characterize the backscattered signal to collagen fibrils of various diameters, how that signal was tuned using both scanning electron microscopy (SEM) and confocal reflectance microscopy (CRM) to create a reverse algorithm, and how that algorithm was used to measure the diameters of fibrils below the diffraction limit. We refer to the spiral response created by these simulations as the FDTD response curve throughout this manuscript.

## 2. Methods

### 2.1. Finite difference time domain

FDTD was used to estimate the shape of the irradiance response curve for varying fibril diameters for a interference confocal reflectance microscope, using the MATLAB platform and a previously constructed FDTD program as a base [27]. Finite–Difference Time–Domain (FDTD) is a type of computational electromagnetics developed by Yee [28] that implements a rigorous solution of Maxwell’s equations through Finite–Difference approximations of the differential equations on a spatial grid. The finite–difference calculation requires small spatial voxels, less than one tenth of the wavelength, as well as small time steps, and thus requires large amounts of memory and time. We have used a two–dimensional computation, which reduces the computational load and is highly appropriate for our cylindrical geometry. The Matlab implementation is discussed by Simon [29]. The code was updated in 2013 by Hollmann [27] who used it for studies of ultrasound modulation of light, and is available at Northeastern University.

Briefly, the index of refraction is mapped in a two–dimensional array, along with the electric and magnetic fields, initialized to zero. A source electric field is introduced into the array as a function of time. and spatial derivatives of the electric field are calculated. Through Maxwell’s equations, the time derivative of the magnetic field is calculated and used to update the current magnetic field. Then the spatial derivative of the magnetic field is processed to update the electric field at the next time step. The electric field is calculated along a line, Hilbert–transformed in time to produce a complex signal and then propagated to the confocal pinhole plane using a Fourier transform, and the squared magnitude is summed over the pinhole to obtain the signal power. The process is repeated for different locations and wavelengths and recorded. Focusing the source field to different locations we could view a 2–dimensional image of the returned power, or by plotting the signals at three wavelengths as functions of fibril diameter, we could generate the FDTD response curve.

Wavelengths of 488 nm, 514 nm, and 561 nm were chosen. A collagen fibril ( $n_{fibril}\approx 1.41$ [26]) was placed in the middle of the simulation, surrounded by a uniform medium. The diffraction limited Gaussian beam was injected at the top of the simulation, focused to a depth near the fibril, and the confocal signal from the fibril was collected, processed as described in the previous paragraph, and the power through the pinhole was recorded. Scanning transversely across the fibril was performed, and the axial beam focused was set in the center of the fibril to save on computation time. Fibril diameters of 10 nm to 1000 nm (in steps of 5 nm) were simulated at all 3 wavelengths.

After the simulations were completed, the response curves were used to create the FDTD response curve described in Section 1. For a given collagen fibril diameter, its 488 nm, 514 nm, and 561 nm measurements are plotted as a point in 3D space (one dimension for each wavelength measurement). Each point on the plot can then be used to compare an unknown confocal reflectance input via global search. The diameter for the point on the FDTD response curve with the smallest distance to the unknown input then becomes that unknowns estimated diameter.

### 2.2. Live-imaging preparation

Type 1 collagen fibrils were stretched across PDMS trenches of approximately 25 µm width, using micro-manipulators and glass micro-needles on the stage of a Nikon TE2000E microscope. This preparation was identical to that of *Siadat et al.*[3,23,30]. Placing the collagen fibrils over the PDMS trench (and therefore not touching the glass) prevented changes in fibril cross section after adhesion to a surface, thereby preserving their original shape and enabling SEM imaging, which is otherwise challenging when fibrils rest on a substrate. 8 total fibrils were prepared and 11 initial imaging sites were identified. This number was reduced to 5 fibril imaging datasets due to some fibrils presenting nonviable imaging properties (sunk into trenches), or destruction of fibrils during SEM imaging. Fibrils were imaged using CRM first then taken to SEM for imaging, as to avoid imaging the palladium coating applied for SEM during CRM imaging.

### 2.3. Confocal reflectance microscopy imaging and data processing

Confocal reflectance imaging of fibrils was done at the Institute for Chemical Imaging of Living Systems (CILS) at Northeastern University. A Zeiss LSM 880 microscope was used at 488 nm, 514 nm, and 561 nm (matching the FDTD setup). Imaging was done dry (in air, $n_{air}\approx 1$ ), to keep conditions between the CRM and SEM imaging as similar as possible. Z-stacks of 6μm thickness (300 images, 20 nm Z spacing per image) were acquired at each wavelength for 5 fibrils. A 20X 0.8 NA Plan Apochromatic objective lens was used to capture the confocal reflectance imaging. The theoretical XY resolution of a microscope is in this configuration is,

(2) $$\text{Abbe Resolution, XY}=d_{x,y}=\frac{\lambda }{2NA}$$

This would result in a theoretical transverse resolution of 305 nm, 321 nm, and 351 nm for 488 nm, 514 nm, and 561 nm respectively. The theoretical axial resolution is,

(3) $$\text{Abbe Resolution, Z}=d_{z}=\frac{2\lambda }{NA^{2}}$$

This results in a theoretical axial resolution of 1.53 µm, 1.61 µm, and 1.75 µm for 488 nm, 514 nm, and 561 nm respectively.

**Fig. 1.**
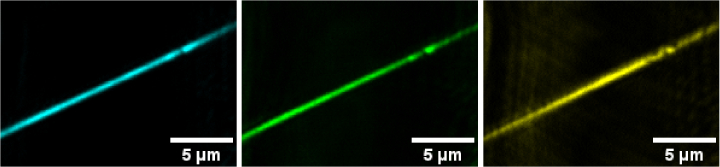
Representative CRM images of a collagen fibril at 488 nm (left), 514 nm (middle), and 561 nm (right).

Fibrils were cropped to avoid to the PDMS trench and ideal focal planes were manually selected using the FiJi/ImageJ software for each fibril Z-stack at each wavelength. We found that no significant tilt was observed over the 25 µm trench, therefore we selected a single ideal focal plane. Z-stacks were then imported into Mathwork’s MATLAB where they were cropped at the selected focal plane and center, as well as shifted if necessary. The approximate cropping window was 10 µm by 15 µm (100 by 150 pixels). Representative images at all wavelengths can be seen in Fig. 1.

To prepare the data for the reverse algorithm after cropping and selecting the appropriate focal plane, the maximum signal along each column was taken for each fibril at each wavelength. Since each image was cropped to 100 x 150 pixels, 150 individual diameter measurements were made per fibril, totaling 750 measurements total for this study.

### 2.4. SEM imaging and diameter estimation

**Fig. 2.**
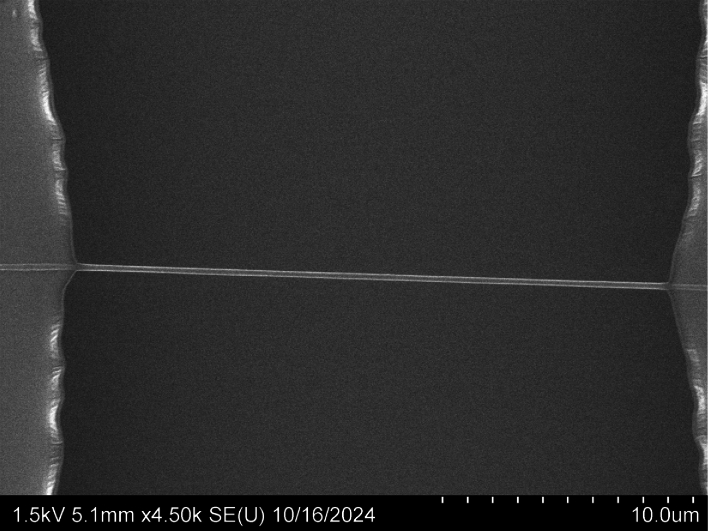
SEM image of a collagen fibril stretched over a PDMS trench.

SEM imaging was done at the Boston Electron Microscopy Center (BEMC) at Northeastern University. The sample was prepared by sputter coating a thin layer (<5 nm) of palladium onto the sample under vacuum. The samples were then imaged using a Hitachi S4800 scanning electron microscope with an accelerating voltage of 1.5 kV and a magnification of 4.5kX. A representative SEM image of a collagen fibril is seen in Fig. 2.

For the SEM images to be registered to the confocal reflectance images, a full field of view SEM image was required (image stitching was not available on this machine, limiting the resolution to the full field of view image of the fibril). In addition, higher resolution images required higher voltage which risked destroying the collagen fibril. As such, the pixel resolution of the SEM images was limited to 11.02 nm.

### 2.5. Data processing workflow

To use the FDTD response curve to estimate the fibril diameters from CRM, the following is needed.

1. Pre-processed CRM data from the three input wavelength images.
2. Scale factors to properly calibrate the FDTD response curve to the microscope counts received in the CRM images.
3. A reverse algorithm for determining the fibril diameter from the calibrated FDTD response curve.

To complete these steps for this manuscript, the following protocol was used:

1. CRM triplets are created by first registering the images via focal plane selection, choosing the fibril center and cropping the PDMS trench to avoid errors, and then selecting the maximum point along each image column for each wavelength. This creates a CRM triplet of three wavelengths for individual points along the fibril, that can be fed to the algorithm for diameter measurement.
2. There are several ways to find the scale factors. First, rigorous photon approximations could be made if information about the microscope is abundant, however this is almost never the case. Second, a calibration reflector could be used in the sample and then used to scale the FDTD response curve. We attempted this with the PDMS, however the surface was too rough for use. To find the proper scale factors in this manuscript, SEM was used to estimate the diameter range and select the appropriate range of the FDTD response curve. Then, ranges of CRM intensity counts were estimated from the previously gathered CRM triplets for all fibrils. Then, the FDTD response curve is scaled to match appropriate minima and maxima given the CRM count ranges and SEM diameter range. The Supplement 1 describes in more detail this scaling process.
3. Our inverse algorithm takes the CRM triplets and searches against the calibrated FDTD response curve to find the closest Euclidean distance, which is then used as the fibrils estimated diameter. This is done via global search. The Euclidean distance is also stored for further analysis of the algorithm and FDTD response curves performance.

## 3. Results

### 3.1. FDTD response curve rescaling

**Fig. 3.**
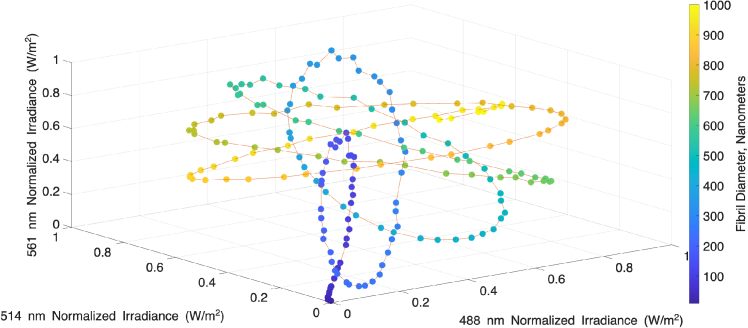
The FDTD response curve, pre-calibration with SEM and CRM results.

The normalized FDTD response curve before calibration with SEM/CRM can be seen Fig. 3.

To crop the range necessary for this study, SEM diameters of the 5 fibrils can be seen in Table 1.

**Table 1.** SEM diameters of five collagen fibrils. For each fibril, there are N = 1901 measurements.

Fibril Name	Average Diameter(nm)	Min-Max(nm)
A	199	181-214
B	223	209-242
C	197	175-237
D	220	175-216
E	192	200-240

**Fig. 4.**
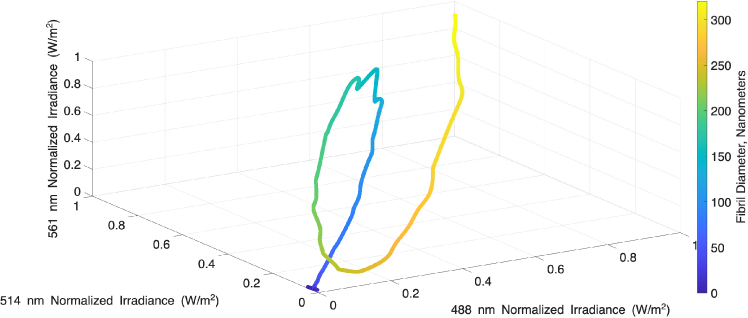
The normalized FDTD response curve, cropped for values between 10-320 nm as suggested by the SEM diameter measurements in Table 1.

From Table 1, we then cropped the FDTD response for fibrils between 10-320 nm in diameter. Values were interpolated between FDTD measurements for a smoother response curve, as seen in Fig. 4.

**Fig. 5.**
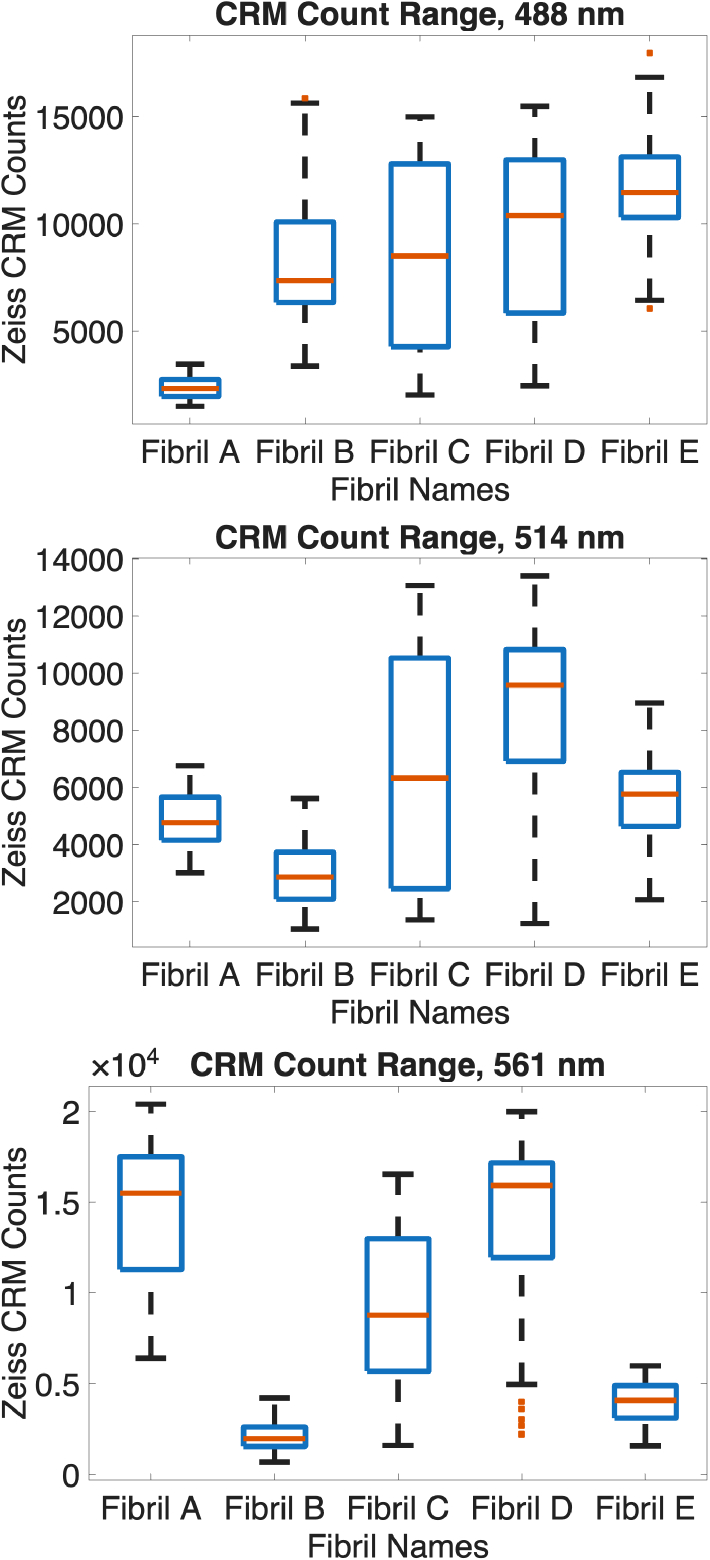
Confocal reflectance ranges for all fibrils, at 488 nm (top), 514 nm (bottom left), and 561 nm (bottom right). Red lines are median, lower and upper box borders are 25-75 percentile respectively. Whiskers extend to include approximately 99.3% of values, with orange dots indicating outliers outside of this range. Each fibril has N = 150 measurements per wavelength.

Using the data gathered in Section 2.3, the box-plots in Fig. 5 represent the confocal reflectance counts generated by the Zeiss LSM 880 for each fibril at each wavelength.

**Fig. 6.**
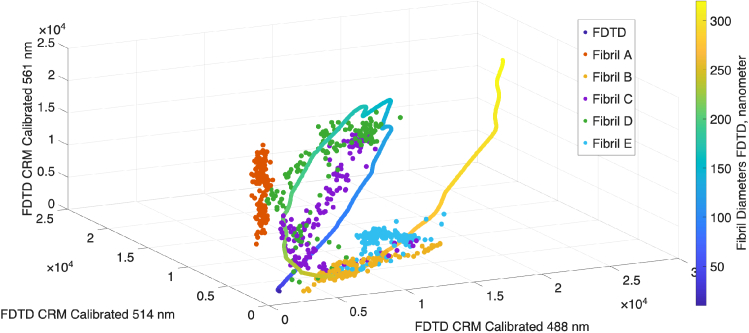
The calibrated FDTD curve alongside all confocal reflectance results.

The final FDTD response curve was then created by scaling the curve according to the ranges in Fig. 5. The final curve is plotted alongside the CRM values in Fig. 6. A more rigorous explanation of this scaling process and range selection is included in Supplement 1.

### 3.2. Inverse algorithm for diameter

**Fig. 7.**
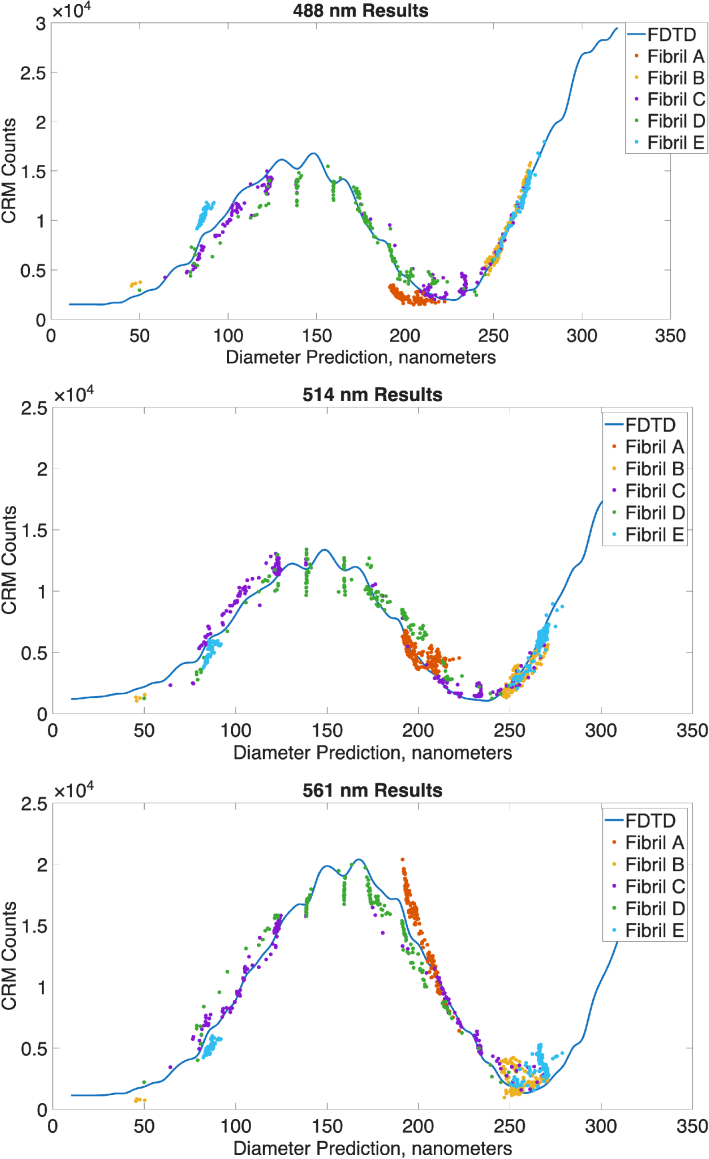
I-CRM diameter measurement results, compared to the calibrated FDTD response curve at each wavelength. These are improved from the plots shown in our SPIE Photonics West proceeding [31]. There are 150 diameter estimations per fibril, making N = 750 for the total dataset.

For each confocal reflectance result gathered in Section 2.3, the diameter was estimated by global search against the calibrated FDTD response curve. The minimum value of this search (closest in the 3D space depicted by Fig. 6) was then used as the estimated diameter for the measured CRM input. All fibrils and their diameters were compared to the FDTD response curve for each of the three wavelengths, seen in Fig. 7.

**Fig. 8.**
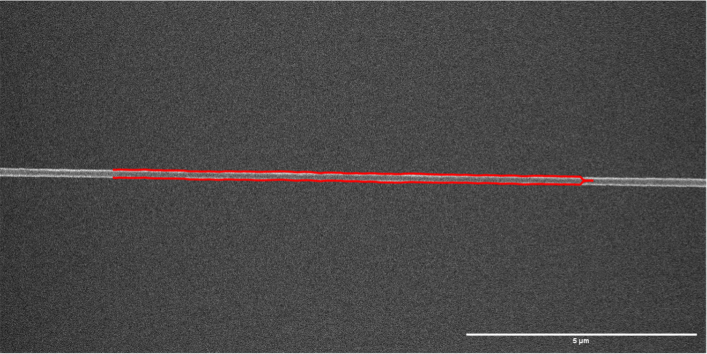
Fibril B SEM image with the predicted I-CRM diameter overlaid with red lines. There are N = 150 measurements in this image.

With registration to the SEM, we then overlaid the predicted diameter from the I-CRM onto the corresponding fibrils SEM image. A representative image of this process can be seen in Fig. 8. This is done via first searching for the correct location on the SEM image via MATLAB, finding the center of the SEM fibril via Gaussian filtering, and then plotting two points above and below the center, along a line perpendicular to the fibril.

## 4. Discussion and conclusion

### 4.1. Collagen fibrils follow the predicted I-CRM FDTD response curve

Figures 7 shows collagen fibrils following the expected shape of the FDTD response curve when calibrated properly. In a non-local interference such as DIC-EIS, larger fibrils would give a response where diameter linearly correlates with measured signal. Figure 7 shows this is not the case, instead following the sinusoidal response described in Figs. 3, 4, and 6.

**Fig. 9.**
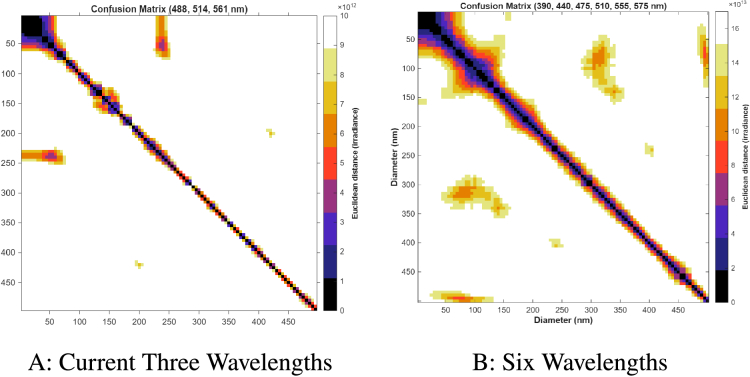
Confusion matrices. A shows the distance in signal space between points representing two different diameters. B shows the same figure for six wavelengths. Note that the distance is in an 6–dimensional space, and that the color scale is increased by approximately a factor of 60.

Because the inverse of one of the quasi–sinusoidal functions for the confocal signal as a function of diameter is not unique, we need multiple wavelengths. Even with multiple wavelengths, inverse of the FDTD response curve in Fig. 3 may not be unique. In Fig. 9(A) we show what we call the confusion matrix. The color represents the distance in the three–dimensional signal space calculated with FDTD, between any two diameters using the three wavelengths of our experiment. Darker colors represent smaller distances. The width of the dark region across the diagonal is a measure of the accuracy of our diameter. Dark values away from the diagonal show regions where the response curve crosses itself and two diameters produce nearly the same triplet of confocal signals. If the distance is small and signals are noisy, the algorithm will find the incorrect diameter, leading to “close–call errors,” such as between diameters around 650 nm and around 450 nm. The number of such close–call regions depends on the chosen wavelengths and the range of diameters included in the FDTD calculation. It can be further reduced by using more wavelengths. Figure 9(B) shows the confusion matrix for six wavelengths. Notice that the color axis is increased by a factor of about 60, indicating that much larger experimental errors can be allowed before close calls will occur. However, the use of six wavelengths doubles the measurement time.

The optimal set of wavelengths among those available on a given microscope depends not only on the avoidance of close calls, but also on the available power, optical throughput of the microscope, and responsivity of the camera. Although the literature on phototoxicity of collagen at the level of monomers and fibrils is sparse, we prefer to avoid the shortest visible wavelengths until we have experiments indicating normal behavior of monomers and fibrils under blue illumination.

### 4.2. I-CRM predictions closely follow the shape of the fibril SEM image

As seen in Fig. 8, the I-CRM diameter predictions closely follow the shape of the fibril for the majority of the points plotted. As mentioned in Section 2.4, the pixel resolution of the SEM was limited to 11.02 nm to capture the field of view (FOV) of the fibril for registration. This means a pixel error of 3-4 pixels in diameter measurement could cause errors above 40 nm, which would correspond to largely different confocal signals on shown on Fig. 7. In addition, the SEM diameter measurement represents a transverse measurement rather than an axial measurement of the diameter, and we believe in certain situations these could be different [30,32]. This makes direct comparison to the I-CRM predictions unreliable, and is a reason FDTD was needed.

What makes the SEM valuable in this case is its ability to show us errors in the I-CRM prediction, such as the rightmost section of the red lines in Fig. 8. The reason for this error is what we are calling “close-call" points on the FDTD response curve. Looking closer at Fig. 6, we see that the majority of points for Fibril B lie in the yellow region ( ∼ 250-300 nm) of the FDTD response curve). However, several points lie closer to the dark blue portion ( ∼ 50 nm). The corresponding SEM image in Fig. 7 confirms that this is likely an incorrect I-CRM prediction.

### 4.3. Quantifying close call points in I-CRM

“Close call" areas refer to the regions on the FDTD response curve (Figs. 4 and 6) where two (or more) regions of the curve are close to overlapping. This would mean two different diameter ranges share CRM signals in all 3 of the excitation wavelengths, making them challenging to distinguish from one another. For example, the dark blue ( ∼ 50 nm) region and green/yellow region ( ∼ 210 nm) of Fig. 4 are particularly close. This is where we believe the overwhelming majority of errors come from in I-CRM. Figure 8 shows this error, and can be further quantified with a boxplot of its predicted I-CRM diameters.

**Fig. 10.**
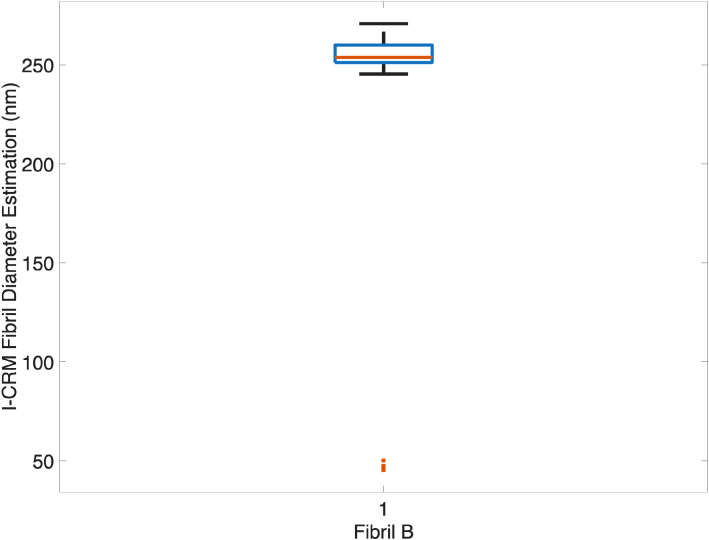
Boxplot of I-CRM diameter predictions for fibril B. The overwhelming majority of values lie around 250 nm, with 4 outliers (quartiles method) at or below 50 nm. These are likely incorrect I-CRM predictions due to close call points.

As shown in Fig. 10, close call errors create a small subset outliers in otherwise consistent datasets of diameter measurements. To quantify the total number of close call errors, we used the SEM overlay technique shown in Fig. 8 for all five fibrils and manually chose obvious inconsistencies with the SEM overlay. These results can be seen in Table 2.

**Table 2.** Close call errors manually counted from SEM overlays of five collagen fibrils.

Fibril Name	Number of Close Call Errors (Median Method Outliers)
Fibril A	0/150
Fibril B	14/150
Fibril C	0/150
Fibril D	1/150
Fibril E	47/150

Total	62/750 (8.3%)

As Table 2 shows, only 8.3% of the total 750 diameter dataset have close call errors, meaning the overwhelming majority of measurements from this method are consistent with the SEM overlay.

Most “close calls” can be eliminated by correcting diameters near discontinuities in the diameter along the fibril. We chose not to make these corrections in this paper in order to demonstrate the ability to measure diameter at a single point. In studies of fibril fatigue or damage, variations in diameter are to be expected so smoothing the data over a large region is not desirable.

Further reduction in the number of “close calls” can be achieved by the use of more or different wavelengths, at the expense of longer measurement time. Many combinations of three or more wavelengths will lead to diameter estimates with good precision and accuracy, and few “close–calls.” Generally, wider spacing of wavelengths will improve accuracy. Wavelengths that result in more detected photons will produce less noisy results. Longer wavelengths imply more photons per Joule, greater optical throughput in the microscope, and higher quantum efficiency in the camera, but lower backscatter. There is some concern regarding phototoxicity, especially with long–time measurements, and longer wavelengths are beneficial for that reason.

### 4.4. Future work

I-CRM provides a new way to measure collagen fibril diameters well below the diffraction limit. It requires no fluorescent staining, is a non-lethal modality, and with a calibration standard (such as a clean piece of glass or similar) could be used without reference to a higher magnification modality such as AFM or SEM.

The next objectives of this work are to decrease the amount of “close call" errors, provide a calibration standard for others to use for normalizing their data to the FDTD response curve, and to use this method in conjunction with confocal fluorescence to track collagen fibril growth in real time with labeled collagen monomer. In addition, we feel this modality has the potential to be used as a full-FOV microscopy method, due to I-CRM’s non-reliance on fiber orientation and ability to make point-by-point diameter estimations. While “close-call" errors could be avoided by averaging points along a fibril, we feel I-CRM’s ability to visualize subtle changes along a fibril should be further explored by pushing the modality towards full-FOV. In addition, we will explore imaging collagen fibrils on glass substrates as this is a common imaging situation with fluorescent experiments. Calibration of the algorithm with microscope parameters and improving signal processing of Z-stacks and focal locations will also be explored, including using fits of intensities along columns instead of choosing single maximum points.

## Supplemental information

Supplement 1 Supplemental document 1, ICRM calibration https://doi.org/10.6084/m9.figshare.32151777

## Data Availability

Code for processing datasets and generating figures is available at [33].

## Supplemental document

See Supplement 1 for supporting content.
