# Supplementary material for: Collagen fibril diameter quantification using interference confocal reflectance microscopy
Source: Biomed Opt Express. 2026 May 11;17(6):2865–79. doi: 10.1364/BOE.596950 (PMC13271222; doi:10.1364/BOE.596950)
Supplement: Supplementary file 1 [file boe-17-6-2865-s001.pdf]

Supplemental Document

Biomedical Optics EXPRESS

Collagen fibril diameter quantification using interference confocal reflectance microscopy: supplement

ERIC HALL,[1,*] SEYED MOHAMMAD SIADAT,[2] JEFFREY RUBERTI,[2] AND CHARLES A. DIMARZIO[1,2,3]

[1]*Northeastern University, Electrical and Computer Engineering, 360 Huntington Avenue, Boston, Massachusetts, USA*
[2]*Northeastern University, Bioengineering, 360 Huntington Avenue, Boston, Massachusetts, USA*
[3]*Northeastern University, Mechanical and Industrial Engineering, 360 Huntington Avenue, Boston, Massachusetts, USA*
[*]*hall.er@northeastern.edu*

This supplement published with Optica Publishing Group on 11 May 2026 by The Authors under the terms of the Creative Commons Attribution 4.0 License in the format provided by the authors and unedited. Further distribution of this work must maintain attribution to the author(s) and the published article's title, journal citation, and DOI.

Supplement DOI: https://doi.org/10.6084/m9.figshare.32151777

Parent Article DOI: https://doi.org/10.1364/BOE.596950

Collagen Fibril Diameter Quantification using Interference Confocal Reflectance Microscopy: supplemental document 1

1. CALIBRATION AND SENSITIVITY ANALYSIS

A. In-depth Calibration Explanation

After the CRM and SEM images were captured, the FDTD response curve needed to be adapted to the dataset. SEM gave us a rough estimate of fibril diameters, as shown in the Table S1.

Fibril Name	Average Diameter(nm)	Minimum-Maximum (nm)
A	199	181-214
B	223	209-242
C	197	175-237
D	220	175-216
E	192	200-240

Table S1. SEM diameters of five collagen fibrils. For each fibril, there are N = 1901 measurements.

SEM predicts fibril ranges from ~175-240 nm. To be cautious and the make sure all of the dataset was encompassed, we chose a range of 10-320 nm to crop the FDTD range.

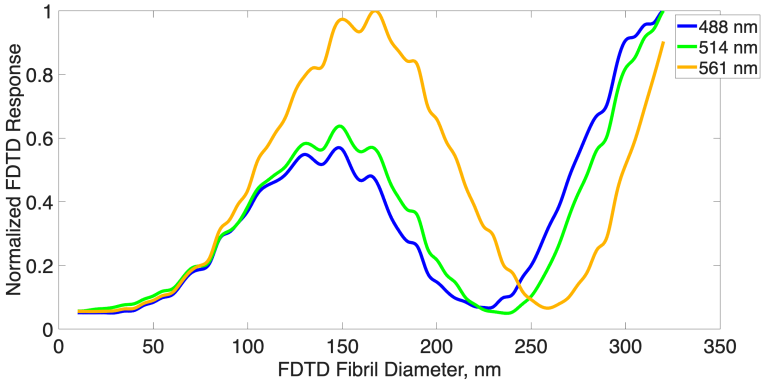

Fig. S1. Normalized FDTD Responses between 10 and 320 nm, as a function of fibril diameter.

The next challenge was rescaling the FDTD curves for each wavelength in Figure S1 into the proper range for the CRM. This was done by using the range of CRM values for each wavelength, ***and assuming the maximum and mins of the CRM ranges corresponded to the first peak and null of the FDTD respectively.***

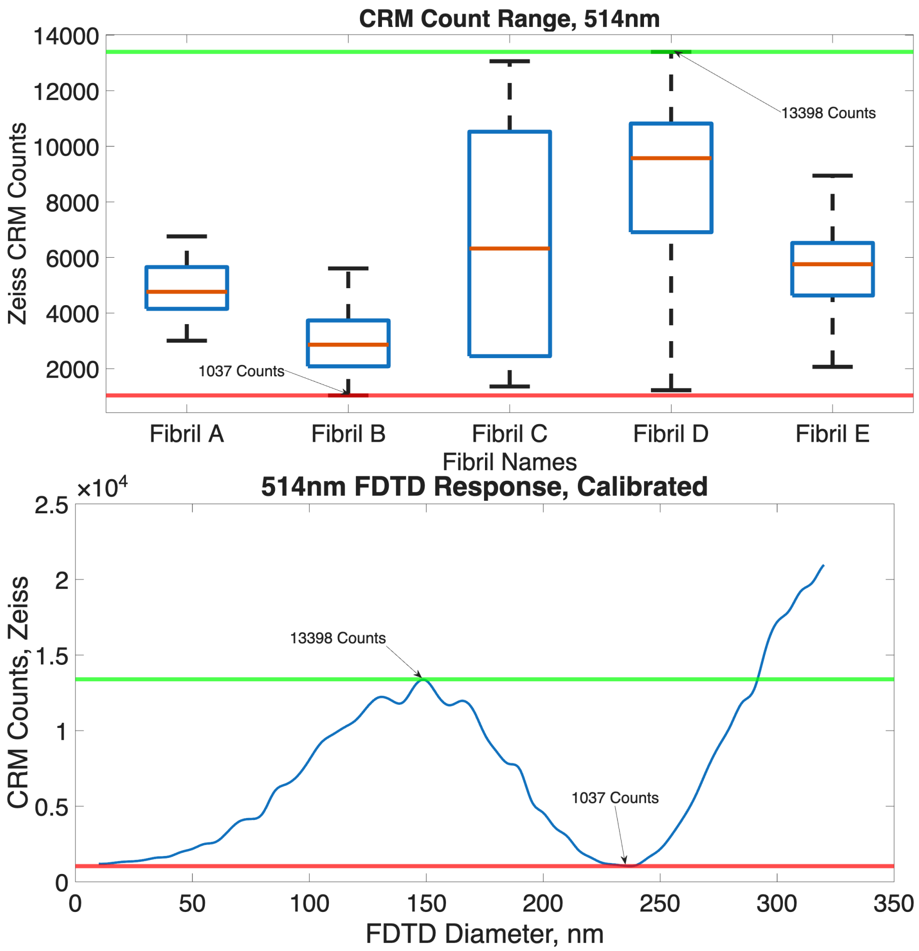

Fig. S2. CRM ranges (top) and using them to calibrate the FDTD response curve (bottom). The green and red lines are identical counts in each plot. Note the maximum of the plot is not scaled to the green line; rather the first maximum peak provided by the FDTD response curve is. This decision was informed by the approximate SEM diameters from Figure S1.

Figure S2 shows this process visually. We first find the $\sim$ 99.6% point of the tallest whisker on the CRM counts, as shown by the top image of Figure S2. This point is highlighted in green. We then take the first maximum of the FDTD response curve and scale it to this green value, as shown by Figure S2 bottom. The minimum is done similarly with the $\sim$ 0.3% value as shown by the red lines in Figure S2, where the first minima of the FDTD is scaled. This is done at 488 nm, 514 nm, and 561 nm separately.

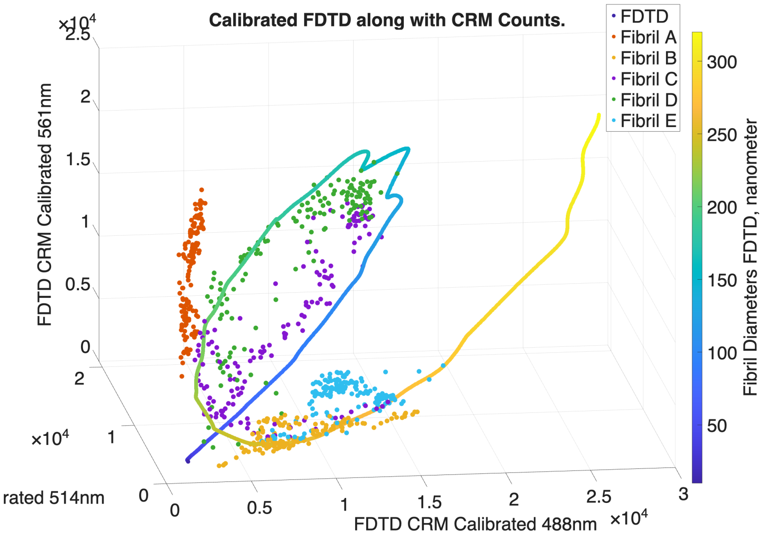

Fig. S3. Calbirated FDTD response curve used to get diameter measurements. The I-CRM diameter is the Euclidean distance point to the FDTD response curve.

We then plot the calibrated FDTD response curve in 3D space (one dimension for each wavelength) along with each point of the confocal data, as shown in Figure S3. The color of FDTD curve corresponds to the diameter. To find the diameter of a fibril using CRM at a given point, its CRM triplet is plotted against the FDTD response curve and the Euclidean distance from each point on the response curve is found. The minimum distance is found for each point, and the diameter from the FDTD response curve is used for that CRM input. In the manuscript, the diameter is used for comparison between fibrils and the SEM. The minimum distance is also stored and used for assessing the performance of the FDTD response curve.

A metric for how well a particular response curve performs is average Euclidean distance for each point used to estimate a fibril diameter. In the case of the calibration used in Figures S2 and S3, the average Euclidean distance was 1994 counts.

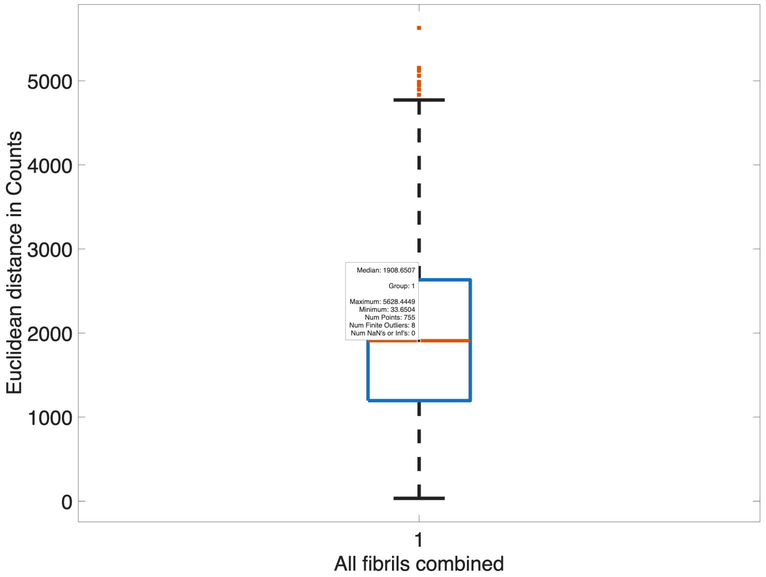

Fig. S4. Euclidean distance of all I-CRM diameter predictions to the original manuscript FDTD response curve.

The following sensitivity analysis shows how the average Euclidean distance changes when

we modify the range gathered from Table S1 and Figure S1, as well as the scaling of the maximum and minimums for each wavelength as shown by Figures S2 and S3.

B. Sensitivity Analysis

B.1. Diameter Range Selection

There are two ways Euclidean distances can be adversely affected in processing. First is incorrect cropping of the FDTD range (eg. 10-320 nm, Figure S1), and the second is incorrect scaling of the CRM ranges to the normalized FDTD (Figure S2). We first present three cropping scenarios: 10-160 nm (original from manuscript), 10-160 nm, and 140-320 nm. For this exercise, we keep the scale factors from the CRM ranges the same.

Range (nm)	Mean	Median	Standard Deviation	Maximum	Minimum
10-320	1994	1909	1066	5628	33
10-160	4863	3816	3075	12,651	523
140-320	2483	2202	1600	8520	19

Table S2. Euclidean distance statistics for different cropping ranges, with the same scaling technique as Figure S2.

As Table S2 shows, the original cropping selection performs the best with the lowest mean, median, standard deviation, and maximum of the Euclidean distance. The minimum is larger for 10-160 nm and negligible (33 vs 19) for 10-320 nm and 140-320 nm. The 140-320 nm does perform noticeably better than 10-160 nm, but this is to be expected considering the SEM informs us from Table S1 that many of the fibril diameters should be in this range. In both cropped cases, the most common error occurs when the closest calibration point in simply the terminal end of the response curve (eg. for 10-160 nm, many points estimating as exactly 160 nm) as shown in Figure S5. This is mitigated by using the original chosen range of 10-320 nm. Fibril E also gives nice insight as to how "close call" errors appear, with the diameter estimation jumping down to 80 nm from the expected 250 nm range of both the original I-CRM calibration and Table S1.

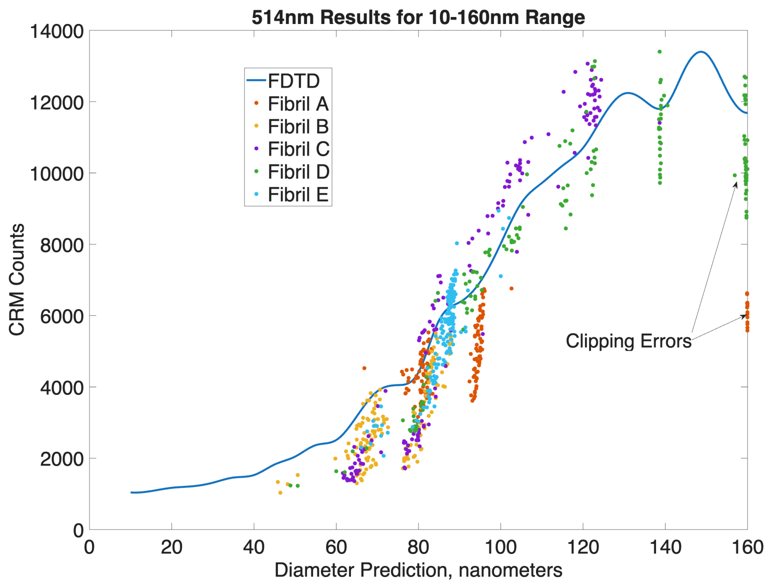

Fig. S5. Clipping errors from incorrectly setting the FDTD response curve diameter range. These cause large increases to average, median, and maximum Euclidean distance numbers as noted by Table S2.

B.2. Wavelength Scaling Selection

For wavelength scaling, there are a few ways to introduce changes to the Euclidean distance statistics. The most challenging in our case was setting the maximum and minimum given by the confocal dataset, after the range of fibrils (10-320 nm in this case) was determined. As mentioned in the Methods, we chose to scale the largest data points from the CRM to the first peak of the FDTD data as shown in Figure S2, inferring from the SEM diameter estimates and the shape of the input data plotted in 3D space (Figure S3). The minimum points were assumed to match the lowest values received from the CRM, matching the valleys of the FDTD response curve.

To test this for the given CRM input data from this manuscript, the upper and lower calibration from Figure S2 (as shown by the green line) was varied between -5000 to 5000 counts from the original calibrations, for each wavelength. For each variation the average and standard deviation of Euclidean distances were plotted, as seen in Figures S6, S7, S8, and S9.

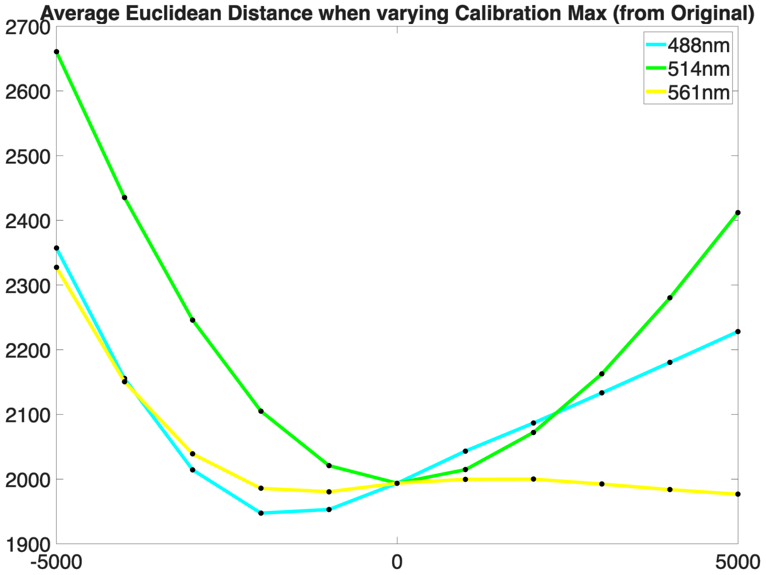

Fig. S6. Average Euclidean distance when varying the maximum offset of the FDTD response curve (green line from Figure S2) for each CRM wavelength.

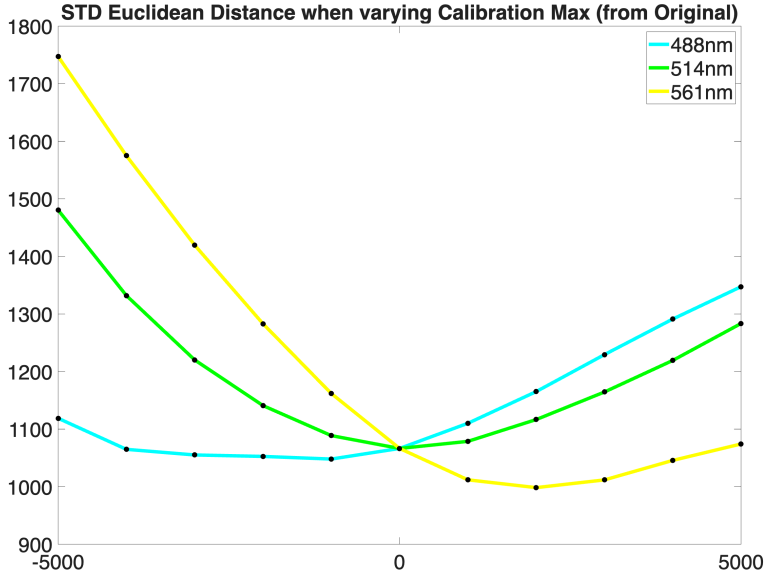

Fig. S7. Standard deviation of Euclidean distance when varying the minimum offset of the FDTD response curve (green line from Figure S2) for each CRM wavelength.

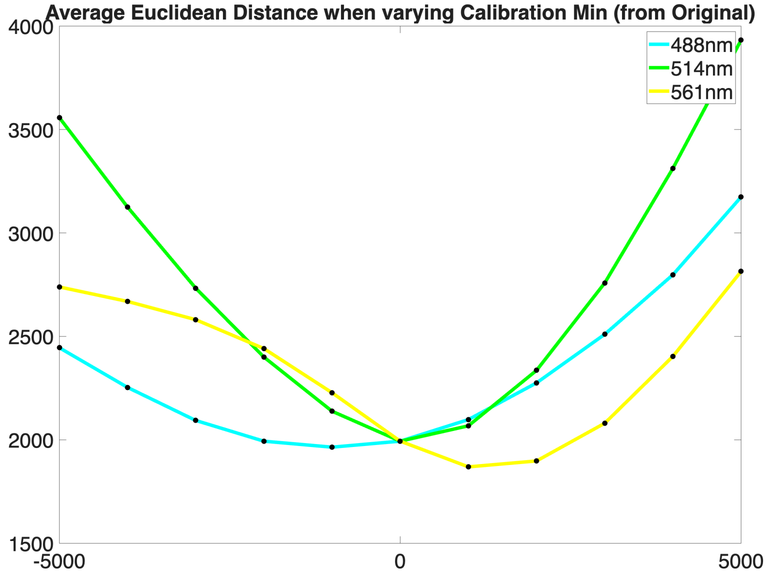

Fig. S8. Average Euclidean distance when varying the minimum offset of the FDTD response curve (green line from Figure S2) for each CRM wavelength.

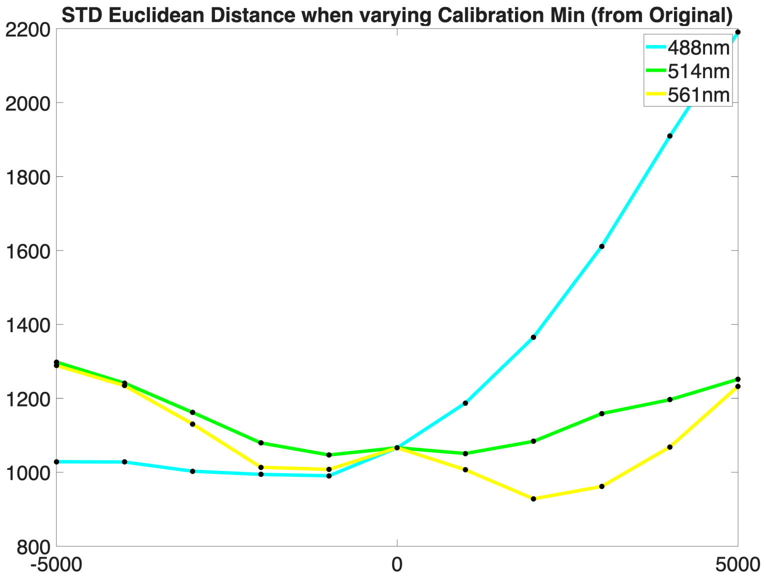

Fig. S9. Standard deviation of Euclidean distance when varying the minimum offset of the FDTD response curve (green line from Figure S2) for each CRM wavelength.

Based on Figures S6, S7, S8, and S9 we feel confident that matching the first FDTD peak for calibration was the correct choice. Wavelengths calibrations can be adjusted slightly, but the change in average and standard deviation of Euclidean distances is not significantly different between the original scaling and ideal. Because the maxima and minima provided by the CRM plots (Figure S2, top) are obvious markers, we feel they are the most appropriate calibration points for this dataset.
